# Hct-A Is a New Actinoporin Family from the Heteractis Crispa Sea Anemone

**Published:** 2014

**Authors:** E. V. Leichenko, M. M. Monastirnaya, E. A. Zelepuga, E. S. Tkacheva, M. P. Isaeva, G. N. Likhatskaya, S. D. Anastyuk, E. P. Kozlovskaya

**Affiliations:** Elyakov Pacific Institute of Bioorganic Chemistry, Far Eastern Branch of the Russian Academy of Sciences, 100 Let Vladivostoku Prosp., 159, Vladivostok, 690022, Russia

**Keywords:** sea anemone, actinoporins, hemolytic activity, lipid membrane conductivity, structural and functional analysis

## Abstract

Several new actinoporin isoforms with molecular weights of 18995.5 to 19398.7 Da exhibiting a high hemolytic activity were isolated from the tropical sea anemone *Heteractis crispa *using a combination of liquid chromatography techniques. The actinoporins were demonstrated to occur as mono-, di-, and trimers in aqueous solutions. The sequences of the genes encoding actinoporins were identified, and the amino acid sequences of the new polypeptides belonging to the Hct-A actinoporin family were obtained. The new acinoporins differ in their isoelectric points, the number and localization of charged amino acid residues at the functionally important N-terminal fragment of the molecule, as well as in the charge of a tetrapeptide (amino acid residues 74–77) involved in an electrostatic interaction with the cytoplasmic membrane. A recombinant actinoporin, rHct-A2, with a molecular weight of 19141 Da, pI of 9.64, and hemolytic activity of 4.0 × 104 HU/mg, was obtained. The conductivity of the ion channels formed by rHct-A2 in the BLM was demonstrated to be similar to that of the native actinoporin from *H. crispa*. The obtained data expand knowledge on the structural and functional relationships of actinoporins and contribute to our understanding of the functioning mechanism of these molecules, which is the basis for the development of compounds with a high biomedical potential. Currently, they are considered as models for obtaining antitumor, antibacterial, and cardiac-stimulating agents.

## INTRODUCTION

The reason for the close attention paid by researchers to sea anemones, marine coelenterates, is their toxins that are complex mixtures of biologically active compounds of a protein nature. Neurotoxins (modulators of Nav, ASICs and Kv ion channels), proteinase inhibitors differing in their structure and mechanism of action, and actinoporins belonging to the α-pore-forming toxin (α-PFT) family are of great interest for future use as pharmacological agents [1-3]. Actinoporins have a unique spatial structure that allows them to exist both in the water-soluble and in membrane-bound states and determines their ability to bind to sphingomyelin- containing membranes and to form ion channels or pores in them [4]. A wide range of pharmacological activities exhibited by these polypeptides, such as anti- tumor, anti-parasitic, dermatonecrotic, and cardiac stimulatory [5-7] activities is associated with the pore formation. It was found that EqtII actinoporin from *Actinia equina *at a concentration of 0.1–1 nM has a cardiotoxic effect, but at higher concentrations it stimulates platelet aggregation [8]. Tenebrosins (actinoporins from *A. tenebrosa*) at low concentrations (~ 10-9 M) were demonstrated to also act as cardiac stimulants [9]. EqtII and Bc2 actinoporins from *Bunodosoma caissarum *[10] were found to be effective anticancer agents affecting fibrosarcoma and glioblastoma [11]. Recently, using HeLa, THP-1, MDA-MB-231, and SNU-C4 cells, we demonstrated that RTX-A actinoporin from *Heteractis crispa*, at nontoxic concentrations, exhibits antitumor effect and suppresses the epidermal-growth-factor-induced tumor transformation of JB6P+Cl41 mouse epithelial cells [7]. This effect was found to be due to the induction of p53-independent apoptosis and inhibition of the activity of the oncogenic nuclear factors AP-1 and NF-κB.

In recent years, α-pore-forming toxins from sea anemones have been used to develop pharmaceutical drugs on the basis of immunoconjugates of actinoporins with ligands, such as monoclonal antibodies, hormones, or growth factors, whose action is directed at the cytoplasmic membranes of tumor and/or parasitic cells [6]. In this regard, investigation of the molecular basis of the actinoporin pharmacological effects seems to be of interest.

This work continues the structural and functional studies of actinoporins. It describes the isolation of actinoporins from the *H. crispa *sea anemone, identification of the sequences of the genes encoding them, production of recombinant analogues, and investigation of some aspects of the mechanism for interaction of actinoporins with biological targets.

## EXPERIMENTAL

Reagents from the following companies were used in the study: Reanal, Hungary; Whatman, UK; ICN Biochemicals, Sigma, Invitrogen, and Thermo Scientific, USA; Fermentas, Lithuania; Merk, Germany; Kriohrom, SibEnzyme, Helicon, and Evrogen, Russia.

**Biological material**

*H. crispa *sea anemones were collected in the intertidal zone of the South China Sea during a scientific expedition on the Akademik Oparin research vessel in 2007. The species of the sea anemone were identified by E.E. Kostina (Institute of Marine Biology, Far Eastern Branch of the Russian Academy of Sciences, Vladivostok, Russia). The sea anemone samples were frozen and stored at –20 °C.

**Isolation and purification of polypeptides**

Preparation of an aqueous extract and precipitation of the total fractions of water-soluble proteins with acetone (63%) were performed as described previously [12]. All manipulations were performed at 4 °C.

Ion exchange chromatography of the polypeptides was performed on a CM-32 cellulose column (2.6 × 50 cm) equilibrated with a 0.01-M ammonium acetate buffer, pH 6.0, with a linear concentration gradient of NaCl (0–0.5 M, total volume of 2 L) in the working buffer. The elution rate was 20 mL/h; the fraction volume was 5 mL.

Gel filtration of the polypeptides was conducted on a Superdex Peptide 10/30 column equilibrated with a 0.1-M ammonium acetate buffer, pH 6.0, using FPLC (AKTA System Pharmacia, Sweden). Elution was performed at a rate of 3 mL/min. The fraction volume was 1.5 mL.

HPLC of the polypeptides was performed on a Nucleosil C18 reversed phase column (4.6 × 250 mm) equilibrated with 10% acetonitrile in 0.1% trifluoroacetic acid using an Agilent 1100 chromatograph (Agilent Technologies, USA). Elution was performed with a concentration gradient of acetonitrile from 10 to 90% in 0.1% trifluoroacetic acid at pH 2.2 for 60 min. The elution rate was 0.5 mL/min. A Concentrator 5301 vacuum concentrator (Eppendorf, Germany) was used for the evaporation of acetonitrile.

The protein concentration was determined by the Lowry method [13]; bovine serum albumin was used as a standard.

**Cloning of actinoporin genes**

cDNA was synthesized based on the total mRNA isolated from tentacles of the *H. crispa *sea anemone [14]. Nucleotide sequences encoding mature actinoporins were amplified using the gene-specific primers 5’-TCGTTACc/aATGATA-3’ (*hct_sign*) and 5’-GATTCTCTATTTGTCTTC-3’ (*hct_notransl*) constructed with the Vector NTI 8 software (Invitrogen, USA) based on the sequences of known actinoporin genes. The primers were synthesized at Evrogen (Moscow, Russia). PCR was performed on a GeneAmp® PCR System 2700 thermocycler (Applied Biosystems, USA) under the following conditions: 94 °C for 5 min; followed by 28 cycles of 94 °C for 30 s, 59 °C for 45 s, and 72 °C for 45 s; followed by 72 °C for 15 min. PCR fragments (650 bp) were isolated from agarose gel with a DNA Extraction Kit (Thermo Scientific, USA) and cloned into pTZ57R/T using a T/A cloning system (Thermo Scientific, USA). Recombinant plasmids were transformed into DH5 *Escherichia coli *cells. Clones were selected using moderately blue selection on a LB medium containing X-Gal and IPTG. The presence of a desired insert in selected clones was determined by colony PCR with standard primers.

**Determination and analysis of nucleotide and amino acid sequences**

Plasmid DNA was isolated by the alkaline lysis method [15]. Determination of the nucleotide sequences of inserts was performed on an ABI3130xl genetic analyzer (Applied Biosystems, USA) [16]. The nucleotide and deduced amino acid sequences were analyzed using the Vector NTI 8 software package (Invitrogen, USA).

**Expression of actinoporin genes**

To generate an expression construct, an actinoporin encoding a DNA fragment was amplified using Vent DNA polymerase (SibEnzyme, Russia) and the gene-specific primers: *hct-a(f)* (5’-GGCTTTAGCTGGTACAATTATCGCGGGTGCA- 3’) and *hct-a(r) *(5’-CCCCAAGCTTAGCGTGAGATCTT AATTT GCAGTAT-3’). To preserve the endopeptidase site and to insert correctly the gene into the pET-41a(+) vector (Novagen, USA), the 5’-end of the forward primer was added with dGMP, and the reverse primer was introduced with the restriction site for HindIII, combined with the stop codon, and with four additional nucleotides for efficient restriction enzyme activity. PCR was conducted under the following conditions: 94 °C for 5 min; then 30 cycles of 94 °C for 30 s, 65 °C for 45 s, and 72 °C for 45 s; followed by 72 °C for 15 min. pTZ57R/T with the *hct-a2 *gene insert was used as the template. The PCR fragment was treated with the HindIII restriction enzyme and cloned into the pET-41a(+) vector at the PshAI and HindIII restriction sites. Recombinant plasmids were isolated and sequenced. Plasmids with the correct insert were used to transform Rosetta (DE3) *E. coli *cells by electroporation on a Multiporator device (Eppendorf, Germany). The transformed cells were cultured in a 2xYT medium containing antibiotics kanamycin (50 μg/mL) and chloramphenicol (34 μg/mL) overnight, after which the culture was grown in a volume of 100 mL until a absorbance A_600_ = 0.5–0.6. To induce expression, IPTG was added (Fermentas, Lithuania) to the final concentration of 0.1 mM and the cells were further grown at 30 0C for 3 h to obtain a fusion protein in soluble form. The cells were then centrifuged (8000 rpm) and washed with a 1× PBS buffer.

**Isolation of recombinant actinoporin**

Cells containing the fusion protein were re-suspended in 1× PBS (1 : 5 by volume) and sonicated on a Sonopuls HD 2070 device (Bandelin Electronic, Germany) to destroy the cell membrane. After centrifugation (10000 rpm), the cell lysate was loaded onto a Ni_2_^+^-CAM agarose, incubated for 10 min (4 0C) with constant stirring for the binding of the fusion protein to the carrier. To remove cell lysate proteins, Ni_2_^+^-CAM agarose with the fusion protein was washed with a buffer solution (50 mM NaH_2_PO_4_, 300 mM NaCl, 10 mM imidazole, pH 8.0) and then with an enteropeptidase reaction buffer solution (20 mM Tris-HCl, 50 mM NaCl, 2 mM CaCl_2_, pH 8.0). The fusion protein was added with enteropeptidase (New England BioLabs, UK) in the amount of 1 enzyme unit per 20 μg of the fusion protein, and the mixture was incubated at room temperature with constant stirring overnight. After sedimentation of Ni_2_^+^-CAM agarose by centrifugation at 3000 rpm, the recombinant actinoporin containing the fraction was collected and incubated with STI agarose to remove enteropeptidase.

**Electrophoretic analysis**

Electrophoresis was performed according to the Laemmli method [17] in vertical plates (9 × 12 × 1 mm) in a 15% polyacrylamide gel in the presence of 0.1% sodium dodecyl sulfate (SDS). Molecular weights were estimated using a PageRuler™ Unstained protein ladder (a set protein markers, 10–200 kDa, (Fermentas, Lithuania)).

**Mass spectrometric analysis**

The molecular weights of the polypeptides were determined on an Ultraflex III TOF/TOF (time of flight) mass spectrometer (Bruker Daltonics, Germany). Time of flight mass spectra was recorded in the linear and reflector modes.

**Hemolytic activity**

The hemolytic activity was detected in mouse erythrocytes in a medium containing 0.9% NaCl. The hemoglobin level in the supernatant was measured spectrophotometrically at 540 nm after preliminary rapid cooling of the reaction mixture and its centrifugation to precipitate erythrocytes and their shadows. The amount of protein causing 50% hemolysis of red blood cells in 1 mL of 0.7% suspension at 37 °C for 30 min was taken as one hemolytic unit (HU).

The results processed according to the variation statistics rules using the MS Office Excel 2007 software package are presented as the mean values of six independent experiments ± standard deviation. The statistical significance of the differences among the indicators was assessed by the ANOVA one parameter test.

**Determination of the N-terminal amino acid sequence**

The amino acid sequence of the recombinant actinoporin N-terminal fragment was determined on a Procise 492 cLC automatic solid-phase protein sequencer (Applied Biosystems, USA) according to the manufacturer’s program using a protein sample on the PVDF membrane. The recombinant protein was transferred from a polyacrylamide gel to a 0.45 μm PVDF membrane (Millipore, USA) in a buffer solution containing 25 mM Tris, 192 mM glycine, 20% methanol, 0.1% SDS, pH 8.3, at 26 V and 60 mA, overnight, using a Mini Trans-Blot® camera (Bio-Rad, USA). The membrane was stained with 0.04% Coomassie Brilliant Blue G-250 in 10% (by volume) glacial acetic acid and then washed free of dye with 50% (by volume) methanol and dried in a thermostat at 37 °C.

**Preparation of bilayer lipid membranes**

Bilayer lipid membranes (BLMs) were formed on the 0.25 mm aperture of a Teflon cup by the Muller method [18] using a 1% solution of monoolein in n-heptane containing predetermined concentrations of sphingomyelin. Aqueous phase: 0.1 M or 1 M NaCl, 10 mM Hepes, pH 7.5. Actinoporins RTX-A (5 ng/mL) and Hct-A2 (50 ng/mL) were added to the aqueous phase until the BLM formation.

**Measurement of BLM electrical characteristics**

The current through the BLM was measured by a VK2-16 high resistance voltmeter-electrometer in the membrane voltage clamp mode using silver/silver chloride electrodes (asymmetry potential of 2.3 mV). Current recording at the amplifier output was performed by means of a KPS-4 potentiometer.

**Homology modeling of actinoporins**

Actinoporins spatial structure models were generated by homology modeling using the SWISS-MODEL web server [19] and Swiss-PdbViewer software [20]. The spatial structure of StnII sticholysin (PDB ID 1GWY) [21] from the *Stichodactyla helianthus *sea anemone, received from the Protein Data Bank, was used as a prototype in constructing the model [22]. Evaluation of the electrostatic properties of the molecular surface in the Amber ff12 force field and visualization of the structures were performed using the MOE software [23].

## RESULTS AND DISCUSSION

According to published data, native actinoporins are usually isolated from the aqueous extracts of whole animals and their further purification is carried out by a combination of various methods of liquid chromatography [4, 12, 24, 25]. In this study, to isolate individual actinoporins, their precipitation from an aqueous extract of the *H. crispa *(*=Radianthus macrodactylus*) sea anemone with acetone and separation of the components of the total protein sample by cation exchange chromatography, FPLC gel filtration, and RP-HPLC were used.

**Fig. 1:**
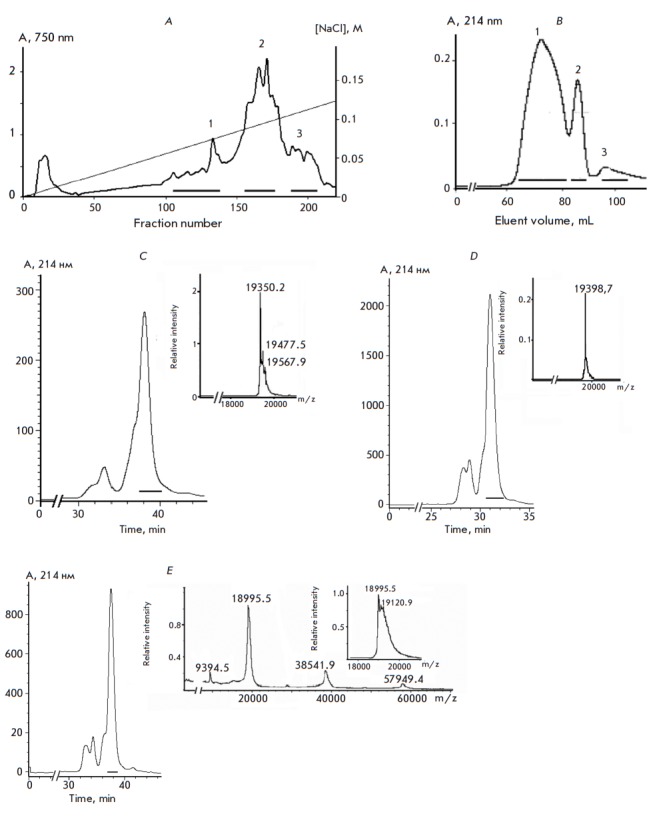
Chromatography of the hemolytic active polypeptides of the sea anemone,* H. crispa*. *A *– Ion exchange chromatography of the total protein preparation on a CM- 32 cellulose column. Boundaries for combining fractions of polypeptides with hemolytic activity are denoted. *B *– FPLC gel filtration of fraction 2 polypeptides after ion exchange chromatography on a Superdex Peptide 10/30 column; boundaries for combining fractions are denoted. *C–E *– RP-HPLC of fractions 1–3 polypeptides, after gel filtration, on a Nucleosil C_18_ column and mass spectra of the obtained compounds

*Figure 1A *presents the elution profile of the total protein sample obtained by chromatography on CM- 32 cellulose. The polypeptides of fraction 2 had a high hemolytic activity, and those of the fractions 1 and 3 had a lower activity. The fraction 2 polypeptides were rechromatographed under the same conditions. Subsequent purification of actinoporins was performed by gel filtration (*Fig. 1B*). In result the hemolitic active fractions containing 50 to 500 μg of the protein were obtained. According to the electrophoretic analysis, these fractions contained polypeptides with a molecular weight of about 19–20 kDa. The polypeptides of fractions 1–3 were subjected to reverse phase HPLC on a Nucleosil C18 column (*Figs. 1C–E*, respectively). Based on this, both fractions of the homogeneous polypeptides (*Fig. 1D*) and fractions containing several polypeptides (*Figs. 1C, E*) with a molecular weight of 18995.5 to 19398.7 Da, according to the mass spectrometric analysis, were obtained. Obviously, actinoporins in the combined fractions are presented as multiple isoforms with very similar physicochemical properties, which is likely the reason for the peak broadening during chromatographic separation of polypeptides.

Previously, from *H. crispa*, we isolated and characterized actinoporins RTX-A, RTX-S, and RTX-SII as well as identified the sequences of the genes and deduced amino acid sequences for 18 actinoporins of the Hct-S family that contain the N-terminal serine residue [12, 14, 25, 26]. The experimental values of the molecular weights of the isolated actinoporins range from 18995.5 to 19398.7 and are consistent with the calculated values for the Hct-S family (from 19338 to 19518 Da) that indicates the existence of many actinoporin isoforms not only at the genomic or transcriptomic, but also at the translational levels. The presence of lower intensity signals in the regions of 38543 and 57950 m/z in the mass spectra demonstrates that actinoporins exist in aqueous solutions likewise in the form of dimers and/or trimers, respectively, which is consistent with the experimental data obtained previously for StnII from *S. helianthus *[27].

It should be noted that the absence of the first two N-terminal amino acid residues is a distinctive feature of the primary structure of RTX-A actinoporin isolated from *H. crispa *(19273 Da) [12]. According to the calculated molecular weights of actinoporins of the Hct-S family (from 19338 to 19518 Da) and the values determined for actinoporins by MALDI TOF mass spectrometry (18995.5 to 19398.7 Da), the existence of another family of actinoporins with smaller molecular weights and, probably, with an alanine residue at the N-terminus of the molecule, may be assumed.

**Cloning of actinoporin genes**

To determine the sequences of the genes encoding mature actinoporins of the *H. crispa *sea anemone, the forward (*hct_sign*) and reverse (*hct_notransl*) primers were constructed. The *hct_sign *primer was constructed on the basis of the signal sequence analysis of known actinoporins, and the gene-specific reverse* hct_notransl *primer was constructed on the basis of previously obtained information on the 3’-untranslated region of *rtx-a *and *rtx-sii *from *H. crispa *[28].

**Fig. 2:**
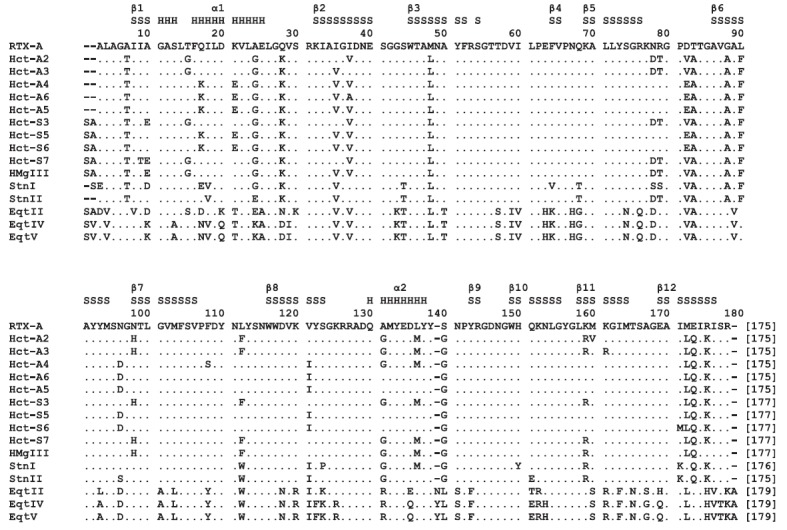
Multiple alignment of actinoporin amino acid sequences. RTX-A, RTX-SII, Hct-As, and Hct-Ss are actinoporins of *H. crispa*; HMgIII is magnificalysin of *Heteractis magnifica *(Swiss-Prot, Q9U6X1); StnI and StnII are sticholysins of *S. helianthus *(Swiss-Prot, P81662, P07845); EqtII, EqtIV, and EqtV are equinatoxins of *A. equina *(Swiss-Prot, P61914, Q9Y1U9, Q93109). Identical residues are marked by points; the length of α-helices and β-strands corresponds to the StnII structure and is denoted by H and S, respectively

With the use of PCR, cloning, sequencing, and analysis of PCR fragments, 17 sequences of the actinoporin genes were obtained, five of which encoded actinoporins of the new Hct-A family (Hct-A2–Hct-A6), and 12 were actinoporins of the Hct-S family established by us previously (*Fig. 2*) [26]. The identity of the nucleotide sequences ranged from 93 to 99%, and that of amino the acid sequences ranged from 88 to 99%. Hct-A2–Hct-A4, Hct-S3, Hct-S5, and Hct-S6 were the most represented.

The calculated molecular weights of the actinoporins of the Hct-A and Hct-S families ranged from 19158 to 19518 Da, which corresponds to the native actinoporins from *H. crispa*, *A. equina*, *S. helianthus*, *H. magnifica*, and *Phyllodiscus semoni *[12, 24-32]. All members of the Hct-A and Hct-S families are highly basic polypeptides; the calculated values of their isoelectric points are in the range of 9.10–9.74, which is typical of actinoporins from *H. crispa, *as well as of the majority of well-known members of actinoporins from other species of sea anemones.

According to the obtained data, a number of actinoporin isoforms encoded by multigene families are synthesized in the tentacle tissue of *H. srispa *as well as the sea anemones *A. equina *[29, 33], *H. magnifica *[34], and* S. helianthus *[24, 35]. Actinoporins differ by single amino acid substitutions (*Fig. 2*), most of which are located in the functionally important amphiphilic N-terminal fragment of the molecule (1–27 aa) that is involved in the pore formation and is associated with the β-core by the highly charged S/KRK30 loop [21, 28, 36]. All actinoporins are characterized by a high conservation of amino acid residues within the aromatic phosphorylcholine (POC) membrane binding site (104–137 aa) as well as the Lys77 residue localized in the loop connecting the β5- and β6-strands (76–79 aa). This residue, as demonstrated for EqtII, is involved in the monomers oligomerization [37].

***In silico *analysis of charged amino acid residues in the area of interaction of actinoporins with the membrane**

To date, it has been established that the molecular mechanism of pore formation is based on the electrostatic attraction between a positively charged actinoporin molecule and the oppositely charged cytoplasmic membrane and on the specific interaction between the POC binding site and the phosphorylcholine head of sphingomyelin [5, 21, 28, 36]. Subsequent conformational rearrangement of the N-terminal fragment of the molecule leads first to its transition to the water-lipid interface and then to its inclusion into the membrane hydrophobic core. The process is accompanied by oligomerization of three, or four, or nine monomer molecules [27, 38-40].

**Fig. 3:**
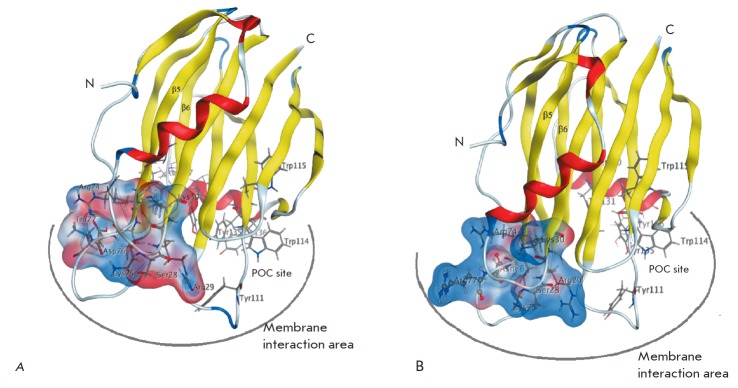
Spatial structure models of Hct-A2 and Hct-A6 actinoporins. The spatial structure models of Hct-A2 (*A*) and Hct- A6 (*B*) actinoporins are presented as a ribbon diagram and colored according to the secondary structure elements. The amino acid residues forming the POC binding site and “pseudorigid” 28SRK30 loop as well as residues with a charged side chain in the loop connecting the β5- and β6-strands are presented as a sticks; the amino acid residues Arg77 and Asn76 of the Hct-A6 actinoporin are presented as a ball-and-sticks. The variable regions of the molecular surface are colored according to electrostatic properties: positively charged residues are in blue, and negatively charged ones are in red. Visualization was performed using the MOE software [23]

To determine the localization of the functionally important sites of actinoporins of the Hct-A family (Hct-A2–Hct-A6), models of their spatial structures were built. The crystal structure of StnII (PDB, 1gwyA) with the highest resolution of 1.71 A (sequence identity ranging from 90.29 to 99.43%) was used as a prototype. The resulting 3D actinoporin structure models contain 12 β-strands, which form the β-core, and two α-helices located at the N- and C-termini of the molecule. The antiparallel β-strands are connected to each other and to the α-helices by different-length loops (*Fig. 3*), which are included into the membrane interface during the pore formation [40]. The RMSD value for 175 Cα atoms of the model relative to the prototype was 0.27 A.

In this study, an analysis of variations in charged residues in the area of interaction of the actinoporins with the membrane was performed. Mapping the molecular surface of these molecules with the changes in their electrostatic properties demonstrated that despite high conservation of the location of charged residues in the structure of the actinoporins, the 74–83 loop connecting the β5- and β6-strands contains a variable region (*Figs. 2 *and *3*). According to cryoelectron microscopy, this loop is localized, like the POC binding site, on the surface of the contacts between the actinoporins and the lipid interface and plays an important role both in the recognition and in interaction with the membrane [21]. Substitution of the neutral Thr residue with the positively charged Arg residue at position 77 and the negatively charged Asp with Asn at position 76 in three members of the Hct-A4–Hct-A6 family was demonstrated to increase significantly the positive charge density in this region (*Fig. 3*). In our opinion, this should lead to a strong electrostatic interaction of both this loop and the adjacent, highly charged, “pseudorigid” SRK loop (28–30 aa) with the membrane surface. Obviously, these are electrostatic interactions that facilitate, in turn, the conformational reorganization of the N-terminal fragment and its subsequent dislocation and integration into the membrane.

**Obtaining of recombinant actinoporin and study of its properties**

A large number of actinoporin isoforms in a single producer species creates certain difficulties in producing homogeneous polypeptides in an amount sufficient in order to conduct structural and functional studies. In order to obtain individual actinoporins, conditions for the expression of their genes in a bacterial system were selected and a scheme of their isolation in the recombinant form was developed.

**Fig. 4:**
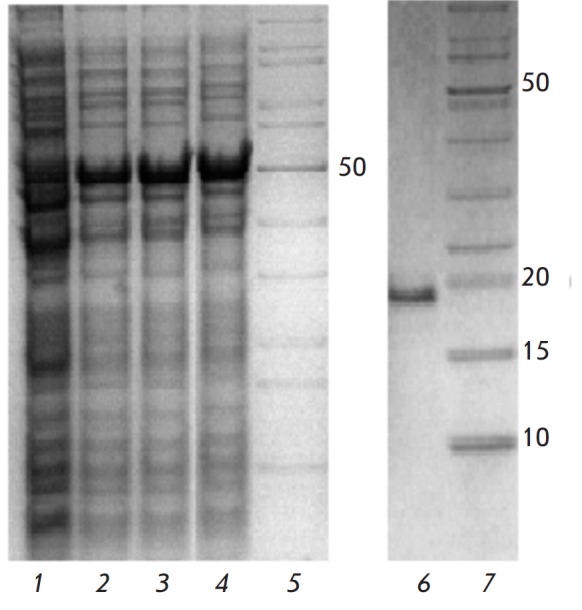
Electrophoregrams of cell lysate proteins after expression of pET41a(+)-*hct-a2 *without addition of IPTG (*1*); after expression of pET41a(+)-*hst-a2 *with addition of IPTG at concentrations of 0.1, 0.5, and 1.0 mM (*2–4*, respectively); and the recombinant rHct-A2 actinoporin (*6*); *5, 7 *– molecular weight markers, kDa

To generate a construct expressing the actinoporin gene, the pET system was chosen; in particular, the pET-41a(+) plasmid vector designed for *E. coli *expression of target proteins fused with a carrier protein, glutathione S-transferase (GST). Based on the sequences of genes encoding mature actinoporins of the Hct-A family, the gene-specific primers *hct-a(f) *and *hct-a(r)* flanking the *hct-a2 *gene at the 5’- and 3’-termini, respectively, were constructed. The recombinant pTZ57R plasmid containing the *hct-a2 *gene was used as a template for PCR. A 550 bp fragment was obtained using PCR that was inserted into the plasmid at the PshAI and HindIII sites. Recombinant plasmids with the desired insert were used to transform the Rosetta (DE3)* E. coli *strain. The recombinant actinoporin was obtained as a GST-fused protein with a polyhistidine tag (GST–His6–rHct-A2). According to the electrophoretic analysis, the molecular weight of the fusion protein was slightly above 50 kDa (*Fig. 4*), which is consistent with the calculated data (~52 kDa). GST–His6–rHct-A2 was also detected in the culture medium, but in smaller quantities. IPTG concentration of 0.1–1.0 mM had practically no effect on the recombinant protein yield. The recombinant rHct-A2 actinoporin with a molecular weight of approximately 20 kDa was isolated from the cell lysate by affinity chromatography under native conditions (*Fig. 4*). The actinoporin yield was 4 mg/L, on average. The N-terminal amino acid sequence (15 aa) was determined by sequencing that fully corresponded to that deduced from the nucleotide sequence. The calculated molecular weight of rHct-A2 was 19141 Da, and the isoelectric point was 9.64. The hemolytic activity of rHct-A2 was 4.0 × 104 HU/mg, which is comparable to that of actinoporins both from *H. crispa *[12, 25, 26] and from other species of sea anemones [4, 5, 9, 24, 29].

**Fig. 5:**
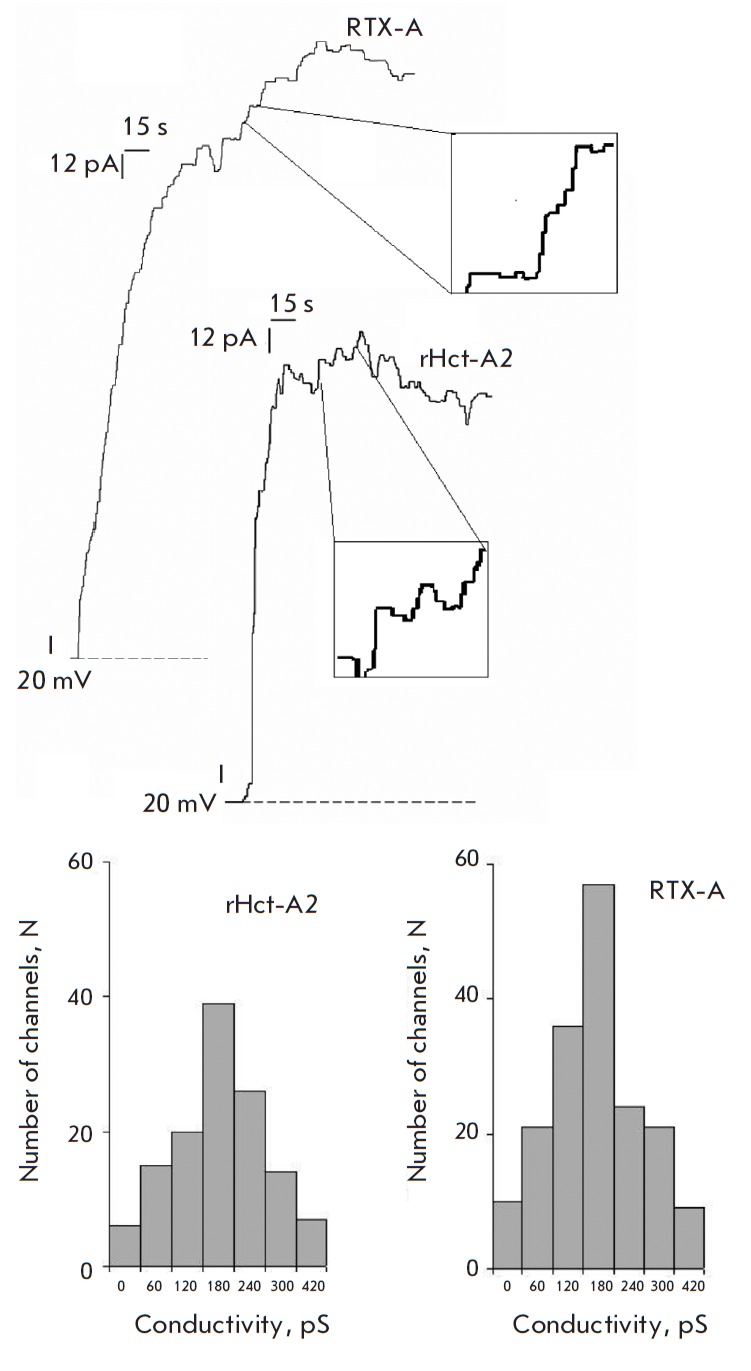
The results of the analysis of the channel-forming activity of actinoporins on the BLM. A – BLM conductivity induced by actinoporins from *H. crispa*: rHct-A2 (50 ng/ mL) and RTX-A (5 ng/mL). The membrane potential is 20 mV. *B *– conductivity histograms of membranes modified with rHct-A2 and RTX-A actinoporins

Based on the analysis of the recombinant actinoporin channel activity on the monoolein : sphingomyelin (1 : 3) BLM, it was found that rHct-A2 at a concentration of 50 ng/mL causes discrete current fluctuations in the membrane, which indicates the formation of conductive structures in the membrane – ion channels (*Fig. 5A*). The most probable conductivity value of the rHct- A2-induced channels in 0.1 M NaCl at pH 7.5 was 180 ± 30 pS, which corresponds to the conductivity of the channels formed by native RTX-A (*Fig. 5B*).

## CONCLUSIONS

According to pharmacological studies of actinoporins, their biological activity is determined by a nonspecific effect that leads to an increase in the membrane permeability, which can stimulate a variety of toxic effects in cells. In fact, an increase in the actinoporin-induced cell permeability leads to profound changes in the morphology of the cell and its organelles, cell fragmentation [5], as well as to an increase in cell size and to cell death [41, 42]. Structural and functional studies of actinoporins, as well as many other toxins, are ultimately aimed at determining their pharmacological activity and therapeutic potential. Currently, α-PFTs of sea anemones are considered as models for the designing of anticancer, antibacterial, and cardiac-stimulating agents [43, 44]. These natural, unique compounds may find successful application as pharmaceuticals (or the basis for developing drugs) in a combined therapy of cancer diseases and/or various cardiac and cytological pathologies.

## Abbreviations

PFT: pore-forming toxins
BLM: bilayer lipid membrane
aa: amino acid residue
GST: glutathione S-transferase
IPTG: isopropyl-β-D-1-thiogalactopyranoside
